# Navigating CAR-T translation in Australia: lessons from an academic program

**DOI:** 10.3389/fmed.2026.1920197

**Published:** 2026-08-10

**Authors:** Rui Ping Amanda Tan, Robyn Walsh, Geoffrey McCowage, Geraldine M. O’Neill, Kavitha Gowrishankar

**Affiliations:** 1 Children’s Cancer Research Unit, The Children’s Hospital Westmead, Sydney Children Hospitals Network, Sydney, NSW, Australia; 2 Faculty of Medicine and Health, School of Medical Sciences, The University of Sydney, Sydney, NSW, Australia

**Keywords:** CAR - T therapy, guidelines, investigator led trial, regulatory approval, translation

## Abstract

Adoptive cell therapies utilizing both gene-modified and unmodified immune cells have revolutionized treatments for cancer and infection. Seven Chimeric antigen receptor (CAR) T cell products have been approved as therapies, providing the momentum to expand their clinical benefit to several cancer types. Many novel receptor-expressing cell therapies remain in preclinical development and require robust clinical testing Translating these into early-phase clinical trials requires navigating manufacturing, quality, and regulatory systems that are often not documented explicitly for academic investigators. This manuscript provides a stage-by-stage roadmap for translating academic CAR T-cell research into an investigator-led Phase I clinical trial in the Australian public sector. It draws on the E2CAR program, the first clinical translation of an EphA2-directed CAR T-cell product into pediatric bone sarcoma, conducted at the Children’s Hospital at Westmead. We present our experience across six stages: construct finalization and vector strategy, manufacturing process development, quality infrastructure, assay development and validation, multi-entity operational coordination, and CTA regulatory engagement and provide recommendations for researchers at each stage. We also present a consolidated program timeline, minimum personnel requirements, and indicative costs to support grant applications and institutional planning. While the operational framework described here was developed specifically for our Health Precinct some of the recommendations we provide are likely to benefit academic groups navigating constraints in comparable settings.

## Introduction

CAR T-cells are genetically engineered immune effector cells that express chimeric antigen receptors targeting tumor antigens and have revolutionized cancer therapy for certain hematological malignancies (1). Currently, the CAR T-cell products approved as therapeutics are for acute lymphoblastic leukemia, lymphoma and multiple myeloma. Only one T-cell receptor expressing cell product has obtained regulatory approval for the treatment of synovial sarcoma. In the effort to expand clinical benefit of cellular therapeutics to several cancers including solid tumors, novel constructs developed by researchers must be translated for use in early-stage clinical trials, which requires investigators to navigate a complex and evolving regulatory and operational landscape.

This is particularly challenging for academic investigators and public-sector institutions in countries with smaller cell therapy markets, such as Australia, where there is a limited domestic Good Manufacturing Practices (GMP) manufacturing ecosystem for cell and gene therapies and reliance on public-sector and/or philanthropic funding. The absence of a dedicated regulatory framework for advanced therapy medicinal products creates additional challenges to clinical translation.

Adherence to the principles of GMP for the manufacture of medicines provides confidence in the quality and purity of the product (2, 3). This is best understood through the 5Ps of GMP manufacturing: Personnel, Premises, Processes, Products, and Procedures (2). Process control, under which Procedures, Premises and Personnel are managed, ensure consistency and quality of the Product. Product control is exerted through additional quality assurance and quality control processes to ensure it is safe.

Biologicals, including CAR T-cell products classified as Class 4 biologicals (4), are subject to a distinct GMP framework under the Australian Code of GMP for Human Blood and Blood Components, Human Tissues and Human Cellular Therapy Products and manufacturing is regulated under the Therapeutic Goods (Manufacturing Principles) determination 2020 (5–9).

Manufacture in a GMP-licensed facility is legally mandated in Australia for products intended for clinical use (6) unless the goods are exempt (10). Schedule 7 (Item 1) of the Therapeutic Goods Regulations 1990 (4) exempts “goods prepared for the initial experimental studies in human volunteers” from the manufacturing licensing requirements of Part 3–3 of the Therapeutic Goods Act 1989 (10).

With the application of the exemption, a first-in-human study of a novel biological can proceed under the Clinical Trial Application (CTA) scheme without a formal GMP licence. However, the TGA nonetheless expects investigators to apply GMP principles and evaluates CTA applications on this basis (11, 12). In Australia, a CTA to the TGA is generally required before a (first-in human) Phase I trial of a biological product can commence.

Where adequate safety data already exist, either from a prior clinical trial or from a trial approved in a comparable regulatory jurisdiction for the same or similar indication, the alternative Clinical Trial Notification (CTN) pathway may instead be used.

This manuscript presents a stage-by-stage account of the E2CAR program from construct finalization through to CTA submission, highlighting challenges faced, and providing practical guidance for investigators. Our aim is to provide operational details that we have adopted from regulatory guidance documents for an investigator-led Phase I clinical trial of a cell therapy product, to guide fellow investigators in their pursuit of initiating early phase trials.

## The E2CAR program: context and scope

The E2CAR product targets EphA2, (Erythropoietin producing Hepatocellular receptor kinase class A2) a cell surface receptor tyrosine kinase overexpressed in multiple solid tumors including pediatric bone sarcomas. EphA2-directed CAR T-cells (E2CAR) have only been previously tested in adult patients with recurrent glioblastoma (13) without sufficiently described GMP controls or formal release testing and hence not suitable to adopt for a CTN application. Moreover, E2CAR has not been evaluated in the pediatric bone sarcoma setting. Although pre-clinical animal data on the efficacy and safety of our E2CAR product has been published (14), no prior clinical safety data exists for this indication and population. The E2CAR program therefore represents the first clinical translation of an EphA2-directed CAR T-cell product in pediatric bone sarcoma, and the regulatory pathway we describe here is an example of how Australian investigators can navigate the TGA framework when bringing a genuinely novel cellular construct into first-in-human testing via the CTA pathway.

### Institutional setting and constraints

The E2CAR trial will be conducted at the Children’s Hospital at Westmead (CHW), a public-sector pediatric teaching hospital within the Sydney Children’s Hospitals Network (SCHN). The Children’s Cancer Research Unit (CCRU) within SCHN is the academic translational research group responsible for the development and manufacture of the E2CAR product, while the Cancer Centre for Children (CCC) at CHW provides the clinical trial delivery and patient care. The trial operates within the constraints typical of academic translational programs: distributed infrastructure across multiple legal entities, reliance on shared facilities, public-sector funding limitations, and the need to coordinate across institutional boundaries.

Critically, manufacturing, quality, sample handling, and clinical delivery are functionally and physically distributed across four entities within the Westmead Health Precinct:

1. SCHN-CHW (Sponsor, clinical trial delivery and patient care)
2. Blood Transplant and Cell Therapies (BTCT) laboratory within the Institute of Clinical Pathology and Medical Research (ICPMR) at Westmead Hospital (governed by NSW Health Pathology), housed within the Western Sydney Local Health District (WSLHD); referred to as WSLHD BTCT in the rest of this manuscript
3. Sydney Cell and Gene Therapy (SCGT) (independent manufacturing quality oversight)
4. Westmead Institute of Medical Research (WIMR) (CAR T-cell product manufacturing premises)

**Figure 1:**
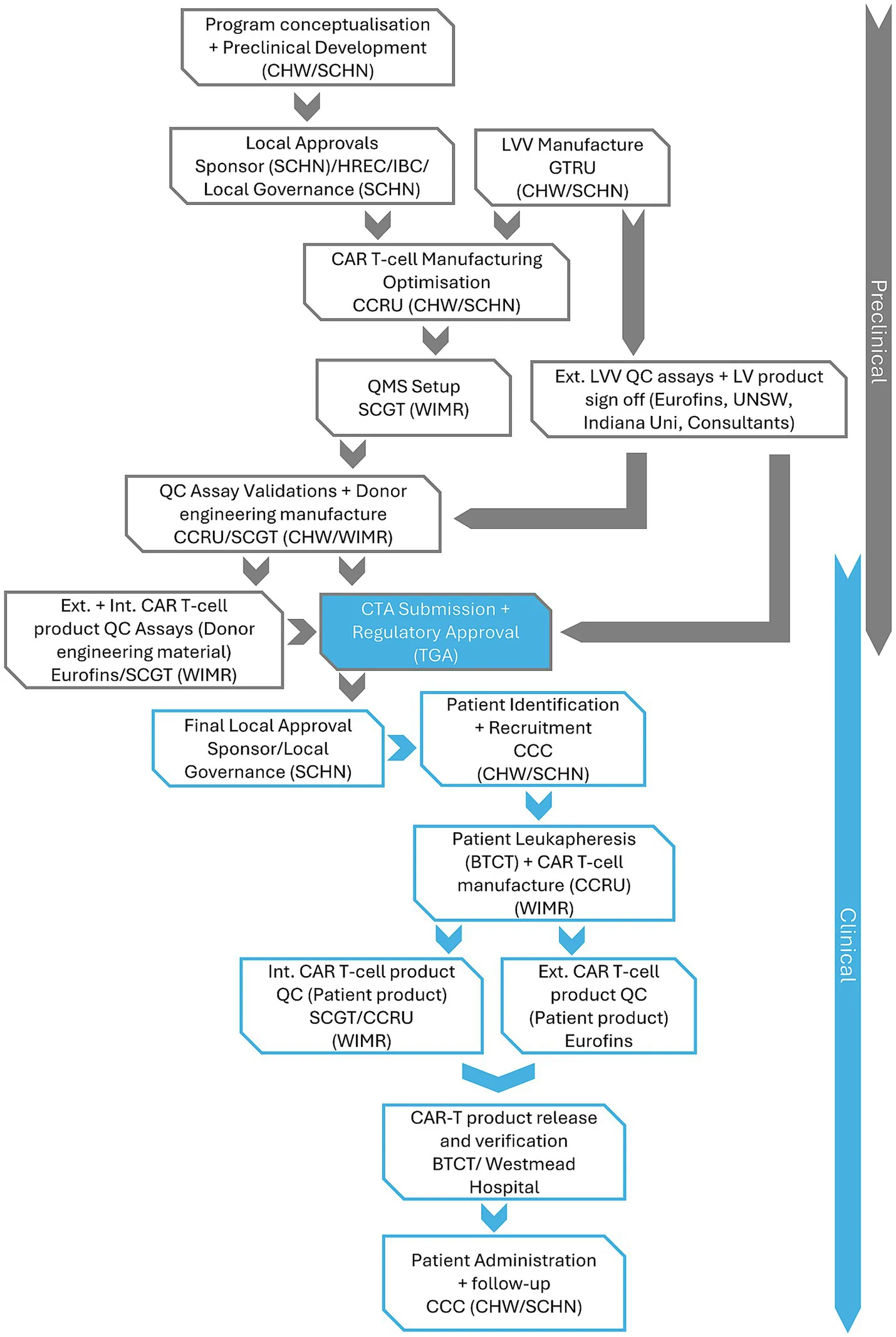
E2CAR program workflow and entity responsibility map. Activities are shown from program conceptualisation (top left) through to patient administration and follow-up (bottom right), with responsible entities and their governing institutions indicated in parentheses. The figure complements the timeline in Figure 2 by illustrating not only the sequence of activities but also the handoffs between entities at each workstream, including external service providers for vector and product quality control. The distributed nature of the workflow, spanning SCHN, CCRU, GTRU, SCGT, BTCT, and multiple external providers, underscores the coordination complexity discussed in Stage 5 and reinforces why early stakeholder mapping and legal agreements are critical-path activities. CCRU, Children’s Cancer Research Unit; CCC, Children’s Cancer Centre; GTRU, Gene Therapy Research Unit; SCGT, Sydney Cell and Gene Therapy; BTCT, Blood Transplant and Cell Therapies; WIMR, Westmead Institute of Medical Research; CHW, Children’s Hospital at Westmead; SCHN, Sydney Children’s Hospitals Network; LVV, lentiviral vector; QC, quality control; QMS, quality management system; CTA, Clinical Trial Approval; TGA, Therapeutic Goods Administration.

Each required separate legal agreement with the SCHN sponsor. The full program workflow and entity responsibilities are depicted in Figure 1.

In addition to the above entities, the Gene Therapy Research Unit (GTRU), a facility within SCHN but organisationally and operationally independent from the E2CAR translational team, was responsible for manufacturing the lentiviral vector. It is important to note that neither the GTRU (vector manufacture), the Human Applications Laboratory (HAL) (site of E2CAR product manufacture), nor SCGT (QA/QC oversight) held formal GMP accreditation at the time of commencement of this work. This is a common reality for academic translational programs and has practical implications for timeline, regulatory engagement, and the extent of qualification testing required. The absence of pre-existing GMP accreditation was a significant contributor to the extended E2CAR timelines discussed in Consolidated Timeline, Personnel and Resource Requirements.

This distributed model, while complex, is likely representative of the reality facing most academic investigators in Australia and similar health systems. The experience described here may be generalizable in comparable settings.

## Stage 1: construct finalization and vector strategy

### Our experience

For E2CAR, we elected to produce the third-generation replication-incompetent lentiviral vector in-house through GTRU rather than source a commercial GMP-grade vector at program inception. The institutional context, distribution of qualification activities, downstream regulatory implications of this decision and generalizable principles arising from this experience are addressed below.

The decision to produce an in-house vector was driven by cost considerations at the small-scale Phase I level and the desire to retain construct flexibility during early development.

Qualification testing was distributed across multiple providers based on accreditation requirements. Sterility, endotoxin, residual plasmid, and replication competent lentivirus (RCL) testing on both End of Production Cells (EOPC) and vector supernatant were outsourced to external GMP-accredited commercial facilities. Host cell DNA and protein contamination testing was outsourced to an external non-GMP-accredited research laboratory. Molecular and functional titre (qPCR for genomic integration; flow cytometry for quantifying target cell transduction), viability, and functionality assays were developed in-house. Ongoing stability testing across the trial duration is conducted by an external service provider. Qualifying the vector required substantial input from multiple members of the translational team and was time- and resource-intensive.

The TGA guidance provided during the pre-submission meeting (Table 1) indicated that the LVV would be treated as a drug substance under TGA-adopted European Medicines Agency (EMA) guidelines, with documentation expected for source materials, cell banks, manufacturing processes, and release criteria, and culture-based (not molecular) mycoplasma testing (15, 16). This generated a substantially heavier Common Technical Document (CTD) Module 3 documentation burden than would be required for a commercial GMP-grade vector supplied with a Certificate of Analysis (CoA) (see Stage 6 for CTD detail) (16, 17).

**Table 1:** General guidance provided during TGA pre-submission meeting for the E2CAR CTA, categorized by domain.

Category	TGA guidance
Submission process	A single, complete CTA submission is preferred.
Lentiviral vector (LVV) requirements	Qualification of the vector (drug substance) to include documentation for source of material used, quality assurance processes for both material and manufacture, including release criteria (in accordance with ICH Q5A(R2), ICH Q5D, TGA Guidance 10) (15, 16, 40). Evidence of Mycoplasma testing via cell culture rather than via molecular methods. Evidence of adventitious virus testing of the cell bank used for vector production (e.g., HEK293T) must be provided, in accordance with ICH Q5A(R2) (16) requirements for viral safety evaluation of cell substrates.
Manufacturing validation	Where pediatric samples cannot be ethically obtained for test runs, healthy donor blood/samples are acceptable.
Pre-clinical and quality data	Provided similar toxicology and quality characteristics are demonstrated, it may be acceptable to submit pre-clinical data generated using different batches of vector. A comprehensive dataset including details of protocol, product characterization and biological activity is required.
Evaluation timeline	Initial evaluation by TGA will take a minimum of 50 business days from receipt of a complete submission, although in practice this is likely to be longer. The full cycle from submission to final approval, including evaluator queries and applicant responses, typically takes 3–6 months.

The items listed reflect recommendations to inform CTA preparation and are not prescriptive requirements for CTA approval.

### Generalizable guidance

Commercially available CAR T therapies utilize either gamma-retroviral vectors (e.g., Yescarta, Tecartus) or lentiviral vectors (e.g., Kymriah, Breyanzi, ABECMA, CARVYKTI). Non-viral transposon vectors are attractive as they reduce costs and are being evaluated in early-phase clinical trials (17).

The make-versus-buy decision for vector is one of the most consequential early choices. In-house vector production offers cost advantages at small scale but demands extensive qualification before it can be used in a Phase I clinical trial. Additionally, use of a research-grade vector developed in a non-GMP-accredited facility may create significant regulatory and development challenges when progressing to Phase II/III clinical trials and product registration. While the TGA (through adopted EMA guidelines), U. S. Food and Drug Administration (FDA), and International Council for Harmonisation (ICH) Q5E comparability framework do not mandate repetition of Phase I, sponsors would be required to demonstrate analytical and potentially clinical comparability between the research-grade and GMP-grade vector products (18–21). If adequate comparability cannot be established, for example, due to insufficient characterization of the original research-grade vector, regulators may require additional clinical studies, which could be equivalent in scope to repeating the Phase I trial. For this reason, use of a GMP-grade vector from the outset of Phase I is strongly recommended to de-risk the clinical development pathway and ensure that early-phase data can support subsequent regulatory submissions without the need for extensive bridging studies. A CoA addressing regulator required qualifications is otherwise provided with commercial GMP-grade vector, but at substantially higher cost.

*Key recommendation*: Finalise the CAR construct and vector source before investing in process development or assay validation of the CAR product. The ideal approach is to obtain vector (research or GMP-grade) via early engagement with a commercial supplier with the capacity for GMP manufacture and scale-up, and with provided evidence of qualification assay completion in a GMP accredited facility. This will minimise the burden of vector qualification testing by the translational team and facilitate use of the same vector testing data for subsequent Phase II/III clinical trial submissions without the need for duplicating Phase I trials.

## Stage 2: building quality infrastructure

Quality infrastructure for Advanced Therapy Medicinal Products (ATMP) manufacture encompasses four key domains: manufacturing premises, equipment qualification, document control, and material procurement and traceability, all required for regulatory confidence in the trial product. Since the TGA’s expectation is that robust GMP compliant quality controls are followed, irrespective of obtaining formal GMP licencing, the quality of various aspects of infrastructure need to be maintained and documented.

Manufacturing premises are classified according to International Organization for Standardization (ISO) standards (grades 0–9), based on airborne particle counts. Cleanrooms are commonly described using grades A–D, where a Grade A environment (ISO 5 equivalent) permits no more than 3,520 particles per cubic meter and a Grade B environment (ISO 7 equivalent) permits up to 352,000 particles per cubic meter (5). A lower-grade manufacturing environment may be acceptable to the TGA where the equipment within it provides sufficient containment; for example, a laminar flow hood classified as Grade A operating within a Grade B room. Environmental monitoring is expected by the TGA irrespective of the cleanroom grade used (5, 22, 23).

Equipment within the manufacturing facility, including incubators, biosafety cabinets, centrifuges, cell counters, water baths, and freezers, requires validation, annual servicing, and routine performance monitoring. Where these facilities are shared between research groups, infrastructure maintenance should be overseen by independent operations teams so that quality control systems are maintained and controlled to prevent product cross contamination and do not require requalification for each new investigator-led trial.

Document control should be established from the outset. Procurement of raw materials, including consumables and reagents involved in any aspect of manufacture and testing, requires a traceable chain of custody to ensure material accountability and safety. Traceability is achieved by documenting the source, origin, quality, sterility, and suitability of each procured material, with a CoA stored alongside the record of receipt. Materials range from tissue culture plasticware (culture flasks, tubes, pipettes, G-rex® vessels) to process-specific reagents such as anti-CD3 beads, cytokines, culture media, freeze media, and buffers, all of which should be manufactured to GMP standards. All suppliers must also be qualified to ensure that materials are handled and shipped in accordance with their specific requirements (e.g., storage at −80 °C or at room temperature), and the condition of each material at receipt and during storage should be logged as part of ongoing material monitoring.

Ideally, supplier and reagent registers, associated documents, and document control are managed using Quality Management System (QMS) software or other regulatory-approved document and inventory management platforms. Quality infrastructure also involves establishing validated qualified assays for product testing addressed in Stage 4.

### Our experience

**Figure 2:**
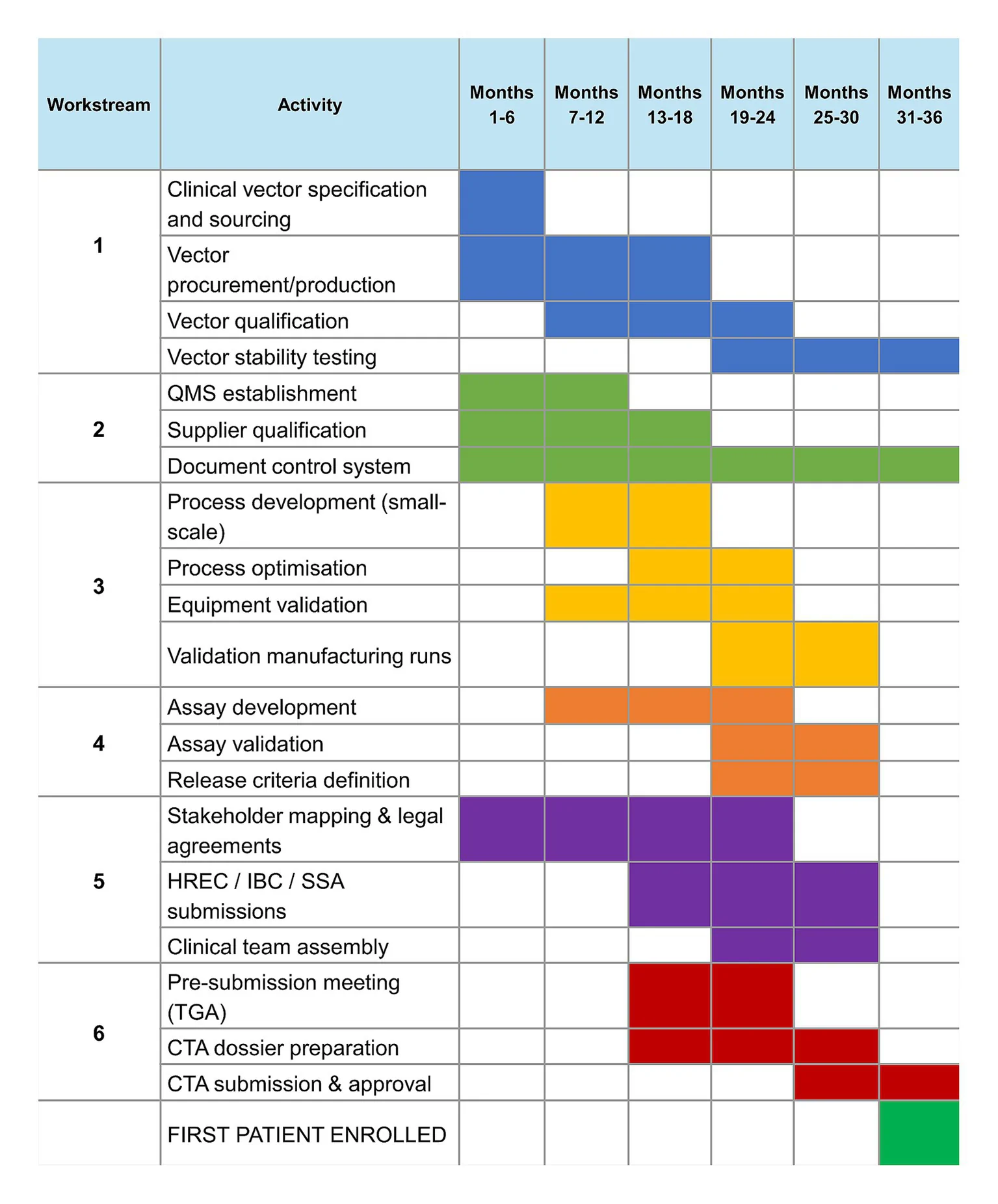
Consolidated timeline for investigator-led Phase I CAR T-cell trial (indicative, based on E2CAR experience). Colored bars indicate active workstream periods. Workstreams overlap significantly; parallel execution is essential. This timeline assumes pre-clinical development (construct design, *in vitro* characterization, animal model studies) has been substantially completed prior to Month 1.

For E2CAR, SCGT served as the independent quality oversight entity responsible for maintaining the shared manufacturing infrastructure at HAL (the Human Applications Laboratory, the site of manufacture) including equipment qualification, environmental monitoring, and document control. We were able to leverage SCGT’s pre-existing QMS, adding documentation for new reagents, materials, standard operating procedures, and suppliers not previously sourced or qualified by them. This arrangement meant that quality systems governing the shared facility did not require requalification for the E2CAR program specifically. QMS systems were also required beyond general infrastructure to include vector qualification and assay validation (workstream 1, 4 and Figure 2).

### Generalizable guidance

Establishing a QMS from scratch is a significant undertaking. Where possible, investigators should seek partnerships with existing quality entities that can provide established QMS frameworks, supplier lists, and document control systems. For investigators without access to quality infrastructure, the minimum suite of quality tools would ideally include supplier registers documenting GMP accreditation or qualification status for each supplier, a reagent and materials list with associated CoAs, document control procedures, and a tracking system for recording material receipt, storage, and use.

*Key recommendation*: Where existing quality infrastructure is available, dedicated personnel for QMS support and documentation management should be allocated from project commencement. Additional team members should be employed for this purpose where investigators are building quality systems from scratch. This person should ideally be independent of the manufacturing team and responsible for maintaining up-to-date CoAs and reference documents required for CTA submission and regulatory audits.

## Stage 3: manufacturing process development

The choice of manufacturing system, open versus closed, is shaped by starting material availability, the number of development optimization runs required, capital cost, and the flexibility needed during process development. In an “open system,” materials and reagents exist within vessels that can be opened, resulting in elevated risk of cross-contamination from the external environment, requiring a Grade A cleanroom for manufacture or equipment equivalences as discussed in Stage 2. A “closed system” is defined as the condition where the product is not exposed to the external environment at any time during production, allowing manufacture in cleanrooms below Grade B. Closed systems such as the CliniMACS Prodigy®, however, require significant upfront capital investment, operate in a fixed “one device per patient” mode that limits process development optimizations, and can be prohibitively expensive for academic institutions reliant on public funding.

### Our experience

The starting material for CAR T manufacture were mononuclear cells collected via apheresis, which serve as the source of T-cells needed for CAR T cell manufacture. In the E2CAR trial, autologous peripheral blood mononuclear cells (PBMCs) will be collected from pediatric patients by BTCT CHW and enumerated. The apheresis specimen is then transported to WSLHD BTCT, which records receipt and release to investigators for transport to the manufacturing facility (HAL). Both BTCT CHW and WSLHD BTCT are accredited by the Foundation for the Accreditation of Cellular Therapy (FACT) and the Joint Accreditation Committee of the International Society for Cell & Gene Therapy and the European Society for Blood and Marrow Transplantation (JACIE) and by the National Association of Testing Authorities, Australia (NATA). NATA is Australia’s national accreditation body for testing and calibration laboratories, operating against the ISO/IEC 17025 standard (24). Comparable bodies in other jurisdictions include the American Association for Laboratory Accreditation (A2LA) and the National Voluntary Laboratory Accreditation Program (NVLAP) in the United States, the United Kingdom Accreditation Service (UKAS) in the UK, and Deutsche Akkreditierungsstelle (DAkkS) in Germany. Investigators planning equivalent programs in other jurisdictions should ensure their apheresis collection and specimen handling facilities hold accreditation from the relevant national body.

Given the constraints on starting material volume due to the target population in this trial, the significant capital cost and fixed “one device per patient” mode of closed systems such as the CliniMACS Prodigy®, and the need for multiple manufacturing process optimization runs, we chose an open-system approach using G-Rex® vessels manipulated within a laminar-flow hood in a Grade B cleanroom at HAL in WIMR, creating a localized Grade A environment. This allowed us the flexibility of optimizing multiple manufacturing parameters such as starting T cell numbers, lentiviral vector quantity used, T cell activation time, and expansion and culture protocols.

Our optimized protocol involves CD4/CD8 magnetic bead-based selection and 48 h activation via anti-CD3/CD28 binding, followed by lentiviral vector-based transduction and expansion in G-Rex® vessels for 12 days in the presence of cytokines IL-7 and IL-15. Having established our optimized protocol, we proceeded to our donor material engineering manufacture to generate a product data package for inclusion in our CTA submission (Stage 6). Across these optimization runs, the key product attributes monitored were transduction efficiency (% E2CAR+), CD4/CD8 composition, post-expansion phenotype and viability, fold expansion, vector copy number, and post-thaw recovery. These parameters served the dual purpose of guiding manufacturing decisions during optimization and identifying the critical quality attributes that would later form the basis of the release specification.

Once the manufacturing process is finalized, ideally, three validation runs need to be performed to demonstrate consistency and reproducibility.

### Generalizable guidance: open vs. closed systems

The higher the grade of clean room, the higher the containment and lower likelihood of introducing contaminants. If grade A facilities are not available, then a laminar flow hood classified as Grade A operating within a Grade B/C room is acceptable with regular documented environmental monitoring results.

Emerging platforms including Lonza’s Cocoon, ThermoFisher’s CTS Rotea (which combines closed-system transduction with flexible expansion), microfluidics-based systems, and automated small-scale bioreactors are expanding the options available, though most are still being validated for clinical-grade manufacture (25, 26).

Non-viral delivery platforms such as MaxCyte and Lonza’s Nucleofector (Amaxa) systems offer electroporation-based alternatives for transposon-mediated CAR gene integration, potentially reducing vector costs and manufacturing complexity compared to viral vectors (27, 28).

*Key recommendation*: For early-phase investigator-led trials requiring multiple optimisation and validation runs, open or semi-closed systems offer critical flexibility. Plan for transition to closed-system or commercial-scale manufacture at Phase II. The choice between open and closed systems should be driven by starting material volume, number of development optimisation runs required, available infrastructure, and budget.

## Stage 4: assay development and validation

Assay development is a critical part of product characterization to establish both product characterization and product safety. These assays undergo validation to demonstrate that the methods utilized are acceptable for their intended use, demonstrating meeting defined acceptance criteria for accuracy, precision, specificity, linearity, range, robustness, and reproducibility. Assay validation therefore involves the systematic and repeated testing of each method, using documented protocols and pre-approved validation plans executed within qualified systems and under the supervision of the quality team, to confirm that the assay produces are consistent and reproducible results are obtained across multiple operators and testing occasions. The validation process is required to establish that the method is fit for its intended use.

Aside from assay validation, equipment and facility validation will also need to be undertaken and can be treated either as part of Stage 2 or alongside assay development if specific equipment is brought in for a newly developed assay not already present within existing infrastructure.

### Our experience

For E2CAR, assays for product release were developed and validated under the guidance of SCGT within their established quality laboratories, with existing equipment and facility previously validated.

Validation plans and protocols were developed by the scientific team, approved by SCGT and carried out by trained personnel on qualified equipment, including the use of regulatory-approved software. Each assay required extensive development and multiple iterations.

### Product evaluation

#### Sterility and mycoplasma testing

Sterility and mycoplasma testing constitute essential safety assays are often outsourced to external accredited testing facilities with established validated methods. Hence, similarly, for E2CAR, testing was outsourced to accredited facilities which were also contracted to handle the validation of these assays.

#### Endotoxin testing

Endotoxin testing may be outsourced or developed in-house. For E2CAR, endotoxin testing was developed in-house alongside the validation of the endotoxin assay.

#### Vector copy number (VCN)

VCN is a product-specific safety assay unique to lentiviral- or retroviral-transduced CAR T products. For E2CAR, we developed digital droplet PCR (ddPCR) assays to determine VCN. ddPCR was chosen over qPCR because Poisson-based absolute quantitation removes standard-curve dependence and improves inter-laboratory reproducibility. As the equipment required for the ddPCR assays had not been established at SCGT prior to our commencement, equipment validation was performed alongside assay validation.

#### Replication competent lentivirus (RCL)

While RCL testing is a critical safety assay for lentiviral vector-based products, emerging practice and recent EMA regulatory update supports a phase-appropriate approach to RCL testing, in which demonstration of negative RCL in the LVV batch, combined with a documented risk assessment for RCL-deficient formation during CAR T-cell manufacturing, may be accepted in lieu of RCL testing on every patient product (CAR T) at batch release (20, 29). Investigators should confirm the acceptability of this approach with their regulatory authority, as expectations may vary by jurisdiction.

### Phenotypic characterization

#### CAR expression

E2CAR T-cell characterization was performed via flow cytometry using an established phenotyping panel (Miltenyi StainExpress™ CAR T Transduction Cocktail) supplemented with our CAR-specific antibody. Product-specific phenotypic characterization will typically need to be developed in-house and tailored to the specific CAR T product. The MACSQuant® flow cytometer used for product characterization had been validated by SCGT previously and as such we only had to validate the characterization assay specific to the E2CAR product.

#### Viability and stability testing

Freeze–thaw viability and stability testing over time were adopted from established assays previously developed by SCGT. Stability testing should be planned to cover the anticipated duration of the clinical trial. Product characterization assays by flow cytometry to determine viability and phenotype were validated as above to provide quality assurance that products kept over a long period of time can be comparatively assessed. The rate control freezer Via Freeze™ by Cytiva was also independently validated to establish its fit for purpose.

#### Efficacy testing

While efficacy is not part of product release as it is not the primary outcome of Phase I trials, we nevertheless demonstrated the cytotoxicity of developed E2CAR cells against osteosarcoma cells in culture. If this were to become a part of product release, then testing for efficacy using validated assays would be required.

### E2CAR release criteria

The release criteria in Table 2 were derived from the optimised manufacturing process development and validation runs described in Stage 3 to ensure that the generated CAR T-cell product meets safety and quality standards for patient infusion.

**Table 2:** E2CAR phase I release criteria.

Parameter	Criterion
Sterility	No bacterial or fungal growth (outsourced)
Endotoxin	<5 EU/mL (in-house)
Mycoplasma	Not detected (outsourced)
Vector copy number (VCN)	<5 copies/cell (in-house)
Phenotype	<2% B-cells and monocytes; >10% E2CAR + (in-house)
Post-thaw viability	>50% (in-house)

Criteria pertaining to patient safety (sterility, endotoxin, mycoplasma, VCN) were defined conservatively based on regulatory expectations (7, 30–32). Product-specific criteria, including the minimum CAR expression threshold of >10% E2CAR + cells, were established at the lower bound of consistent performance observed across process development runs using multiple healthy donors, ensuring that the release specification reflects achievable product quality rather than optimal performance from any single run.

### Generalizable guidance

Sterility, mycoplasma, and endotoxin testing constitute the main safety testing assays and can often be outsourced to external accredited testing facilities with established validated methods. Product-specific assays such as VCN, post-thaw viability as well as phenotypic characterization, efficacy, and stability testing, will typically need to be developed in-house and tailored to the specific CAR T product.

Release criteria pertaining to patient safety (sterility, VCN, mycoplasma, endotoxin) should be defined conservatively. Criteria relating to product characteristics (phenotype, CAR expression, CD4/CD8 ratio) may be product- and indication-specific and should be determined based on preclinical data and clinical protocol requirements.

*Key recommendation*: Begin equipment supplier engagement and assay development in parallel with manufacturing process development (stage 3). Equipment qualification and assay validation are among the longest lead-time items. Each instrument used in release testing (flow cytometer, ddPCR system, cell counter, rate-control freezer, etc) must be independently qualified and certified to GMP standards.

## Stage 5: operational assembly and governance

Although presented here as Stage 5 for narrative clarity, operational assembly and governance activities must commence at or near the beginning of the translational program, running in parallel with Stages 1 and 2 (see Consolidated Timeline).

### Our experience

The E2CAR trial sponsored by SCHN required coordination across multiple internal and external stakeholders within and beyond the CHW. The institutional relationships and project progression between these entities are summarized in Figure 1. Establishing internal stakeholder agreements as outlined in the introduction was substantially more time-consuming than anticipated. The burden is likely now reduced for subsequent investigator-led cell and gene therapy studies conducted across the same network of entities.

Legal agreements were also required between the sponsor and external stakeholders who included suppliers for reagents, equipment, instruments, outsourced assays, and consultancy firms engaged to confirm acceptability of the lentiviral vector.

Ethics and governance approvals include Human Research Ethics Committee (HREC) approval for the clinical trial and associated documentation, Institutional Biosafety Committee (IBC) approval for E2CAR, given it is gene-modified (with the precise classification depending on the nature of the product and method of CAR gene introduction), and local Site-Specific Assessment (SSA) from affected hospital clinical departments authorizing CHW as the trial site.

We were fortunate in procuring funding for a dedicated Clinical Trial Project Coordinator, who serves the role of a Project Manager (PM) and a part-time Clinical Nurse Specialist. The PM has been essential for ensuring smooth integration between the manufacturing team and clinical delivery team, and for meeting SCHN and TGA governance requirements. The PM will also ensure strict adherence to the approved clinical trials protocol.

### Generalizable guidance

Investigators should map all required institutional stakeholders at the earliest possible stage and identify which entities require independent legal agreements with the trial sponsor. Governance approvals (HREC, IBC, SSA) can run in parallel but have dependencies, for example, IBC classification may affect the HREC submission, and SSA requires an approved protocol.

Where the required infrastructure is not concentrated within one institution, investigators should consider whether apheresis collection, sample handling, or quality oversight can be performed at other accredited sites, with appropriate transport and chain-of-custody documentation overseen by the quality team.

Gene therapies involving genetically modified organisms are also regulated by the Office of the Gene Technology Regulator (OGTR) under the Gene Technology Act 2000. However, since 2011, clinical trials of gene-modified somatic cell therapies, including CAR T-cell products, may be exempt from OGTR licensing if the final cell product does not contain replicating virus or produce infectious agents (33, 34). For lentiviral vector-transduced products such as E2CAR, this exemption is likely to apply provided RCL testing confirms no replication-competent virus is present in the final product. This would be demonstrable with LVV testing discussed in both Stage 1 (our LVV experience) and Stage 4 (product evaluation). For where the exemption does not apply, an OGTR licence must be obtained before the trial commences, a process that can take up to 150 business days (35). Regardless of whether a licence or exemption applies, IBC endorsement is required and should be sought early in the planning process.

*Key recommendation*: A dedicated Clinical trials coordinator or Project Manager is an absolute requirement. Whether funded independently or provided through existing institutional trials teams, this role is essential for managing the interface between manufacturing, quality, governance, and clinical delivery. This role needs to be budgeted for from the outset.

## Stage 6: CTA submission and regulatory engagement

The mandatory CTA submission for a Class 4 biological with no prior evidence of testing, consists of two parts (36). Part 1 is the CTA application form for the supply of unapproved therapeutic goods, accompanied by the supporting technical dossier that can be presented using the CTD as a technical dossier framework. Part 2 is the notification of trial commencement, lodged within 28 days of commencing supply at each site, and includes signed certifications from the sponsor, the Principal Investigator, the approving HREC, and the institution authorizing the conduct of the trial. Evidence of local governance and IBC approval is also maintained by the sponsor as part of the site-specific authorization process.

The TGA assesses CTA applications for product quality, safety, labeling and chain of custody, and adherence to Therapeutic Goods Orders (TGOs) and TGA-adopted ICH, EMA and FDA guidelines for biologicals. Although the TGA has not mandated a CTD format for the technical data supporting Class 4 biological CTA applications, it is a standardized dossier framework developed by the ICH and adopted by the TGA in 2004 for the registration of medicines on the Australian Register of Therapeutic Goods (ARTG) (37). The CTD is organized into five modules: Module 1 is region-specific, while Modules 2 to 5 follow an internationally harmonized structure.

The TGA-adopted ICH guidelines (Q5A(R2), Q5B, Q5D, M3(R2)) and the EMA guideline on quality, non-clinical and clinical requirements for investigational ATMPs describe the kinds of data expected within the quality, nonclinical and clinical sections of a technical dossier, and indicate what should be considered according to product and trial risk rather than prescribing specific assays (15, 16, 20, 38, 39). Investigators should refer to TGA Therapeutic Goods Order (TGO) and TGA-adopted ICH, EMA and FDA guidelines to determine what should be included in a CTD-structured dossier for biological products. Table 3 maps each CTD module to the manuscript stages where the relevant data is generated, providing investigators with a practical cross-reference between the dossier framework and their translational program.

**Table 3:** CTA dossier structure mapped to manuscript stages, showing CTD module content and key data sources.

CTD module	Content	Manuscript stage	Key data sources
Module 1	Administrative and regional information	Stage 5	Sponsor details, HREC, IBC, SSA approvals, proposed labeling
Module 2	Summaries of Modules 3–5	Cross-stage	Quality overall summary, nonclinical overview, clinical summary
Module 3	Quality (CMC)	Stages 1–4	Vector qualification and characterization, manufacturing process description and validation, release assay methods and validation, release criteria, stability data
Module 4	Nonclinical study reports	Stage 1	Pre-clinical efficacy and safety data, *in vivo* (murine) studies, biological activity, cytotoxicity data
Module 5	Clinical study reports	Stages 5–6	Clinical protocol, investigator’s brochure (where applicable for Phase I)

### Our experience

We requested a formal pre-submission meeting with the TGA to obtain guidance on our quality and manufacturing strategy.

The pre-submission meeting provided general recommendations that informed our approach to vector documentation, manufacturing validation planning, and assay selection. Table 1 summarizes the key guidance received, organized by category. The TGA evaluators also recommended that investigators refer to the regulatory guidance documents listed in Table 4. The recommendations provided are not absolute requirements for CTA approval but reflect the TGA evaluators’ general guidance for investigators preparing CTA submissions for novel biological products.

**Table 4:** Regulatory guidance documents recommended by TGA evaluators during pre-submission meeting for the E2CAR CTA.

Document	Scope and relevance
ICH Q5A(R2) (16)	Viral safety evaluation of biotechnology products derived from cell lines of human or animal origin
ICH Q5D (15)	Derivation and characterization of cell substrates used for production of biotechnological/biological products
TGA Guidance 10 (40)	Adventitious agent safety of medicines, including testing of cell banks for mycoplasma, endogenous and exogenous viruses
ICH M3(R2) (39)	Non-clinical safety studies for the conduct of human clinical trials and marketing authorization for pharmaceuticals
EMA ATMP guideline (20)	Guideline on quality, non-clinical, and clinical requirements for investigational advanced therapy medicinal products in clinical trials
FDA CAR T guidance (18)	Considerations for the development of chimeric antigen receptor (CAR) T-cell products: Guidance for Industry

### Generalizable guidance

As the product will vary in each case, it is recommended that independent guidance/pre-submission meetings with the TGA is sought as soon as possible. Investigators should structure their data collection around the CTD framework from the outset of the translational program. Planning data generation, record-keeping and document control to align with CTD modules from the beginning substantially reduces the effort required for dossier preparation and minimizes the risk of data gaps discovered late in the program (Table 3). As highlighted in the introduction extension of early stage to later stages will require full GMP manufacturing in a licenced facility. There should be no assumption that any exemption will be granted, for example Schedule 7 exemptions pertaining to conventional medicines.

Investigators who choose to generate their own vector should factor the associated documentation burden into project planning and resource allocation from the outset which is described in Stage 1 and detailed in Tables 1, 4.

Investigators should also be aware that any change in vector batch during a clinical trial will be treated as a variation requiring regulatory assessment, and that a vector batch produced for a Phase I trial under non-GMP conditions cannot be re-used for subsequent GMP-licensed studies. These constraints reinforce the recommendation in Stage 1 to engage a commercial GMP-grade vector supplier from the outset where feasible.

Gene-modified cell therapies, including CAR T-cell products, are also subject to regulation by the OGTR; the regulatory requirements and exemption pathways are addressed in Stage 5.

*Key recommendation*: Engage the TGA via a pre-submission meeting as early as feasible. Their feedback will shape your quality strategy, manufacturing approach, and required assays. This meeting can occur while assay validation and governance approvals are still in progress. From the outset, structure data collection and documentation to align with CTD module requirements (Table 3), and plan for vector documentation at drug-substance level of detail (Tables 1, 4).

## Consolidated timeline, personnel, and resource requirements

The following consolidated resources are based on the E2CAR experience and are intended as indicative estimates for academic investigators planning similar translational programs. Actual timelines and costs will vary with institutional context, product complexity, existing infrastructure, and regulatory requirements.

### Consolidated timeline

The timeline below reflects the clinical translation pipeline and does not include preliminary pre-clinical activities, which for E2CAR included *in vitro* characterization and murine model studies (14). Pre-clinical efficacy and safety data form a core component of the CTA dossier (CTD Module 4; Table 3) and should be substantially completed before entering the translational stages described here.

Figure 2 presents a Gantt-style overview of the six parallel workstreams involved in our program. These correspond to the stages described through this manuscript but are presented here as concurrent workstreams to reflect the parallel execution required in practice. Total program duration from commitment to clinical translation through to first patient enrolled is estimated at 30–36 months, with significant overlap between workstreams. The critical path typically includes vector sourcing and qualification (Workstream 1), CAR T-cell manufacture optimization and establishment of quality management documentation and practices (Workstreams 2/3), CAR T-cell release assay validation (Workstream 4), HREC and local regulatory approvals (Workstream 5), and CTA preparation, submission and approval (Workstream 6).

Key observations from our experience: (1) Workstreams 1 and 2 must commence simultaneously; vector strategy and quality infrastructure need to be established in parallel. (2) Manufacturing process development (Workstream 3) and assay development (Workstream 4) should run in parallel, with assay validation typically extending beyond process optimization. (3) Legal agreements and governance approvals (Workstream 5) frequently take longer than expected and should commence early. (4) The CTA dossier (Workstream 6) can be assembled progressively as data from earlier workstreams becomes available.

Investigators with access to pre-existing GMP-accredited facilities may achieve shorter timelines in workstreams 1, 2 and 4. Conversely, investigators who are working with non-accredited facilities should anticipate timelines at or beyond the upper end of these estimates and should factor the additional burden of qualification testing into their project planning and grant applications.

### Minimum personnel requirements

Table 5 outlines the minimum personnel required for an investigator-led Phase I CAR T-cell trial, with estimated full-time employment (FTE), the stages at which each role is most critical, and the regulatory training each role requires. Total team FTE at peak activity is approximately 3.5–5.0 FTE.

**Table 5:** Minimum personnel and estimated FTE for investigator-led Phase I CAR T-cell trial.

Role	Est. FTE	Stages	Function	Required training
Chief/Principal Investigator	0.2–0.3	1–6	Strategic oversight of the translational program, funding acquisition, clinical protocol and trial design, participant recruitment and medical care, trial staff oversight, data integrity and analysis, and publication.	ICH-GCP training and currency (mandatory for named investigators) Human Research Ethics training (HREC requirement) GMP awareness (strategic oversight level) Biosafety awareness for gene-modified organisms (IBC requirement)
Manufacturing Scientist	1.0	1–5	Investigator Brochure development, CAR manufacturing process development, optimization, validation, CAR T product manufacture	Comprehensive GMP principles training Cleanroom gowning and aseptic technique qualification Biosafety training: handling gene-modified organisms and lentiviral vectors (IBC/OGTR requirements) Equipment-specific operation and maintenance training QMS documentation and deviation management training
Quality Manager	0.5–1.0	2–6	GMP adherence oversight, QMS establishment, document control, QA oversight, manufacturing staff education, TGA audit readiness; ideally independent of manufacturing	Comprehensive GMP training (PIC/S and TGA-adopted guidelines) QMS administration and audit training ATMP regulatory requirements (TGA, EMA-adopted guidelines) Document control and change management training Risk management training ICH-GCP awareness
Quality Control Scientist	0.5–1.0	4–5	CAR release assay development, validation, release testing; equipment-specific training	GMP principles training Equipment-specific qualification and validation training (flow cytometer, ddPCR, cell counter, endotoxin reader) Regulatory-approved software training (data acquisition and analysis) Assay validation methodology (accuracy, precision, specificity, linearity, range, robustness, reproducibility) Biosafety training for handling gene-modified material
Quality Officer	0.5	1–4	Vendor qualification, quality document generation and management, material management	GMP awareness training Supplier qualification procedures training QMS documentation and record-keeping training Material handling, storage, and cold-chain management training
Project Manager	0.5–1.0	5–6	Overarching coordination of the translational program from pre-clinical translation through to clinical trial conduct, including timeline and budget management, clinical protocol and IB development, regulatory and governance submissions (TGA, HREC, IBC, SSA), service provider and inter-entity agreements, manufacturing/QC/clinical team liaison, safety reporting, and GCP compliance oversight	ICH-GCP training and currency (mandatory) Human Research Ethics training TGA regulatory framework (CTA/CTN pathways, CTD structure) Institutional governance procedures (HREC, IBC, SSA) Safety reporting requirements (SUSAR, DSUR) GMP awareness (manufacturing–clinical coordination level)
Trial Nurse (Part-time)	0.2–0.5	5–6	Trial participant coordination/care, apheresis scheduling/process/specimen transport, clinical team educational material development/delivery, data collection	ICH-GCP training and currency (mandatory for clinical trial staff) Human Research Ethics training Protocol-specific training (apheresis, conditioning, CAR T infusion, CRS/ICANS grading) Safety reporting and adverse event documentation training Biosafety awareness for gene-modified cell products at point of administration
Regulatory Affairs Advisor	0.1–0.2	1, 6	Pre-submission meeting preparation, dossier review. Required where the trial team lacks regulatory affairs expertise; typically engaged on a consultancy basis.	Specialist expertise assumed; currency with evolving TGA expectations for ATMPs and biologicals is essential

CRS, cytokine release syndrome; DSUR, development safety update report; GCP, Good Clinical Practice; GMP, Good Manufacturing Practice; HREC, Human Research Ethics Committee; IBC, Institutional Biosafety Committee; ICANS, immune effector cell-associated neurotoxicity syndrome; ICH, International Council for Harmonisation; OGTR, Office of the Gene Technology Regulator; PIC/S, Pharmaceutical Inspection Co-operation Scheme; QMS, quality management system; SSA, Site-Specific Assessment; SUSAR, suspected unexpected serious adverse reaction; TGA, Therapeutic Goods Administration.

Documented training is a GMP requirement and forms part of the evidence assessed during TGA evaluation of the CTA dossier. All personnel named on the clinical trial delegation log (including the Principal Investigator, Project Manager, and Trial Nurse) must hold current International Council for Harmonisation – Good Clinical Practice (ICH-GCP) certification, and all staff involved in manufacturing and quality activities require GMP training at a level appropriate to their role. Biosafety training relevant to gene-modified organisms is additionally required for personnel handling lentiviral vectors or CAR T-cell products, in accordance with IBC and OGTR requirements. Training records should be maintained within the QMS from commencement of the program, as they may be requested during TGA audit or as part of regulatory queries following CTA submission.

Where existing shared infrastructure is available (e.g., SCGT for quality management, institutional clinical trials units for coordination), several of these roles may be partially covered by existing staff, significantly reducing the dedicated FTE required. Conversely, investigators building from scratch should expect to need full FTE at the upper range of these estimates.

### Indicative cost ranges

Table 6 provides order-of-magnitude cost estimates for the major workstreams of an investigator-led Phase I CAR T-cell trial with 3–6 patients over approximately 3 years. These estimates are based on Australian costs as of 2026 and are intended to support grant applications and institutional planning rather than serve as precise budgets.

**Table 6:** Indicative cost ranges for investigator-led Phase I CAR T-cell trial (AUD, 2026 estimates).

Workstream	Lower estimate (AUD)	Upper estimate (AUD)	Key variables
Viral vector (GMP grade, commercial, vector QC)	$150,000	$500,000+	Scale, number of patients treated, titre, supplier; in-house production; lower upfront but higher time cost
Cleanroom access/facility fees	$15,000 p.a.	$25,000 p.a.	Shared vs. dedicated; existing vs. new build; grade B vs. grade A
GMP-grade reagents & consumables	$40,000	$200,000	Per manufacturing campaign; cytokines, beads, media, cell expansion vessels
Equipment qualification & validation	$20,000	$80,000	Flow cytometer, ddPCR, cell counter, incubators; service contracts
QC assay outsourcing (sterility, mycoplasma)	$10,000	$40,000	Per batch; accredited lab fees; turnaround time affects schedule
Quality management (QMS, document control)	$15,000	$50,000	Software licenses, personnel time, audit preparation
Personnel costs (total team, 3 years)	$600,000	$1,500,000	Largest cost; FTE mix, salary scales, institutional overheads
Regulatory/CTA submission	$10,000	$30,000	TGA fees, regulatory consultant, dossier preparation
Governance (HREC, IBC, SSA)	$5,000	$15,000	Institutional fees; amendment costs
Clinical delivery (per patient)	$5,000	$20,000	Apheresis, conditioning, infusion, monitoring; hospital-dependent
ESTIMATED TOTAL (3-year Phase I, 3–6 patients)	$900,000	$2,500,000+	Highly variable; shared infrastructure reduces significantly

Ranges reflect variability based on institutional context, product complexity, and existing infrastructure.

Personnel costs represent the largest proportion of total expenditure (typically 50–70%). Investigators should note that vector procurement from a commercial GMP supplier, while expensive, can significantly reduce downstream time and costs by avoiding duplication of qualification assays. While optimization of the assays can be performed using less expensive research grade reagents, validation and generation of data for the CTA submission will require the use of GMP reagents and consumables to closely mirror that to be used in the clinical trial.

Although these costs are significant, the investment extends beyond the immediate clinical trial. The workforce expertise and institutional infrastructure established through an investigator-led cell therapy program positions the institution to support subsequent early-phase trials with reduced setup costs and shorter timelines. For institutions where shared quality infrastructure already exists, total program costs may be substantially lower than the upper estimates above. Conversely, where new GMP-capable infrastructure must be built from scratch, costs may exceed these estimates. Investigators should also consider that the institutional capability developed through an initial program represents a durable national asset for advancing cell and gene therapy clinical trials, reducing the translational gap for novel therapeutics that are unlikely to attract commercial development.

### Limitations

This account reflects the experience of a single-center study (E2CAR). Our example is specific to an autologous, lentiviral vector-transduced CAR T-cell product for a rare pediatric solid tumor with limited starting material. Several pivotal choices, including the open- versus closed-system decision, assay selection, and release specifications, are shaped by this product and treatment population. While challenges faced at other academic institutions may be similar, some of the guidance provided may not be directly translatable to those seeking to use non-viral platforms, closed or automated manufacture or adult and hematological indications. The consolidated timelines and costs are indicative, estimates drawn from a single program and are further shaped by our starting position without pre-existing GMP accreditation. Programs that can access accredited infrastructure will have a different timeline and cost estimate. Finally, while the quality principles described are internationally harmonized, the regulatory pathway itself is specific to Australia, which in most parts follow EMA guidelines. Investigators elsewhere will need to map our approach to their local framework.

This review is intentionally limited in scope, to focus on offering the most practical value to academic investigators initiating an early-phase cell therapy trial in a resource-limited public-sector setting.

## Conclusion

It is possible to initiate investigator-led Phase I clinical testing in small institutions or public-sector and philanthropy-funded research hospitals by following and adhering to the core principles of GMP manufacturing, even if a TGA-licensed facility for manufacturing is not available.

The key principles that emerged from our E2CAR experience were, finalizing the CAR design alongside manufacturing process development as well as initiating assay validation in parallel is crucial for timely translation. Additionally, early stakeholder mapping is equally important, as legal agreements and governance approvals in multi-entity public-sector environments take substantially longer than investigators typically expect. Finally, engaging with the regulatory agency as early as possible in the form of a pre-submission meeting is highly beneficial as it will guide the application process including documentation required.

We hope that the processes and timelines outlined here will encourage fellow researcher investigators to develop and test novel cell therapies in-house, especially for rare conditions that are often underserved by the commercial sector due to limited market potential. Investigator-led programs represent an important avenue for narrowing the translational gap for these patients and in many instances have delivered meaningful clinical options. Sustaining and expanding this impact, however, depends on academic effort being matched by coordinated support from, funding bodies, regulators, and institutional infrastructure providers.

## Glossary

ARTG: Australian Register of Therapeutic Goods
ATMP: Advanced Therapy Medicinal Products
BTCT: Blood Transplant and Cell Therapies
CAR: Chimeric Antigen Receptor
CCC: Cancer Centre for Children
CCRU: Children’s Cancer Research Unit
cGMP: current Good Manufacturing Practise
CHW: Children’s Hospital at Westmead
CoA: Certificate of Analysis
CTA: Clinical Trial Approval
CTD: Common Technical Document
CTN: Clinical Trial Notification
ddPCR: digital droplet Polymerase Chain Reactions
EMA: European Medicines Agency
EOPC: End of Production Cells
EphA2: Erythropoietin producing Hepatocellular receptor kinase class A2
FACT: Foundation for the Accreditation of Cellular Therapy
FDA: Food and Drug Administration
FTE: Full-Time Equivalent
GMP: Good Manufacturing Practise
GTRU: Gene Therapy Research Unit
HAL: Human Applications Laboratory
HREC: Human Research Ethics Committee
IBC: Institutional Biosafety Committee
ICPMR: Institute of Clinical Pathology and Medical Research
ISCT: International Society for Cell & Gene Therapy
ISO: International Organization for Standardization
JACIE: Joint Accreditation Committee of ISCT
LVV: Lentiviral Vector
NATA: National Association of Testing Authorities
OGTR: Office of the Gene Technology Regulator
QA: Quality Assurance
QC: Quality Control
QMS: Quality Management System
qPCR: Quantitative Polymerase Chain Reaction
RCL: Replication Competent Lentivirus
SCGT: Sydney Cell and Gene Therapies
SCHN: Sydney Children’s Hospital Network
TGA: Therapeutic Goods Administration
VCN: Vector Copy Number
WIMR: Westmead Institute for Medical Research
WSLHD: Western Sydney Local Health District

## References

[1] Arunachalam AK Grégoire C Coutinho de Oliveira B Melenhorst JJ. Advancing CAR T-cell therapies: preclinical insights and clinical translation for hematological malignancies. Blood Rev. (2024) 68. doi: 10.1016/j.blre.2024.101241, 39289094

[2] Pharmaceutical Inspection Convention Pharmaceutical inspection co-operation scheme (PIC/S) (2023). Guide to good Manufacturing Practice for medicinal Products Part I. Available online at: https://picscheme.org/docview/6606 (Accessed May 14, 2026).

[3] Pharmaceutical Inspection Convention Pharmaceutical Inspection Co-Operation Scheme (PIC/S) (2023). Guide to good Manufacturing Practice for medicinal Products Part II. Available online at: https://picscheme.org/docview/6607 (Accessed May 14, 2026).

[4] Department of Health Disability and Ageing. (2025) Therapeutic Goods Regulations 1990. No. 394. Australia. Available online at: www.legislation.gov.au

[5] Pharmaceutical Inspection Convention Pharmaceutical inspection co-operation scheme (PIC/S). (2023) Guide to good Manufacturing Practice for medicinal Products Annexes. Available online at: https://www.gmp-compliance.org/files/guidemgr/pe-009-17-gmp-guide-xannexes.pdf (Accessed May 14, 2026).

[6] Blood Tissues and Cellular Therapy Products Group/Office of Manufacturing Quality. (2013) Australian Code of Good Manufacturing Practice for human Blood and Blood Components, human Tissues and human cellular Therapy Products. Australian Government Department of Health and Ageing Therapeutic Goods Administration. Available online at: https://www.tga.gov.au/sites/default/files/manuf-cgmp-human-blood-tissues-2013.pdf (Accessed May 14, 2026).

[7] Department of Health TGA. (2022). Regulation of Advanced Therapies. Available online at: https://www.tga.gov.au/products/biologicals/human-cell-and-tissue-products/overview/regulation-advanced-therapies (Accessed May 19, 2026).

[8] Department of Health TGA. (2024) Review of the Clinical Trial Approval (CTA) Scheme. Available online at: https://www.tga.gov.au/products/unapproved-therapeutic-goods/access-pathways/clinical-trials/review-clinical-trial-approval-cta-scheme#background-and-resources-for-the-current-cta-scheme (Accessed May 14, 2026).

[9] Department of Health Disability and Ageing. (2025) Therapeutic Goods (Manufacturing Principles) 2020. Australia. Available online at: https://www.legislation.gov.au/F2020L00864/latest/text (Accessed May 14, 2026).

[10] Department of Health Disability and Ageing. (2025). Therapeutic Goods Act 1989. No. 89. Australia. Available online at: www.legislation.gov.au (Accessed May 14, 2026).

[11] Department of Health TGA. (2006) Australian Clinical trial Handbook. Available online at: https://www.tga.gov.au/resources/guidance/australian-clinical-trial-handbook (Accessed May 19, 2026).

[12] Department of Health TGA (2020) PE009, the PIC/S Guide to GMP for Medicinal Products TGA Interpretation and Expectations for Demonstrating Compliance. Canberra:Therapeutic Goods Administration.

[13] Lin Q Ba T Ho J Chen D Cheng Y Wang L . First-in-human trial of EphA2-redirected CAR T-cells in patients with recurrent glioblastoma: a preliminary report of three cases at the starting dose. Front Oncol. (2021) 11:694941. doi: 10.3389/fonc.2021.694941, 34235085 PMC8256846

[14] Hsu K Middlemiss S Saletta F Gottschalk S McCowage GB Kramer B. Chimeric antigen receptor-modified T cells targeting EphA2 for the immunotherapy of paediatric bone tumours. Cancer Gene Ther. (2021) 28:321–34. doi: 10.1038/s41417-020-00221-4, 32873870 PMC8057949

[15] ICH. (1997) Derivation and Characterisation of cell Substrates Used for Production of Biotechnological/Biological Products Q5D Current Step 4 Version International Conference on Harmonisation (ICH). Available online at: https://database.ich.org/sites/default/files/Q5D%20Guideline.pdf (Accessed May 14, 2026).

[16] ICH. (2023) Viral Safety Evaluation of Biotechnology Products Derived from cell lines of human or animal origin Q5A(R2) Final Version. International Conference for Harmonisation (ICH). 10185809

[17] Funasaka C Naito Y Kubota H Ishiguro Y Fuse N Wakabayashi M . Trial in progress: phase I study of non-viral gene-modified CAR-T cell therapy for malignant solid tumors expressing EPHB4 receptor (CARTiEr). Front Oncol. (2025) 15:1633324. doi: 10.3389/fonc.2025.1633324, 40842575 PMC12364634

[18] FDA-CBER. (2024) Considerations for the Development of Chimeric Antigen Receptor (CAR) T Cell Products Guidance for Industry. Available online at: https://www.fda.gov/media/156896/download (Accessed May 19, 2026).

[19] ICH. (2004). Comparability of Biotechnological/Biological Products subject to Changes in their Manufacturing process Q5E Current Step 4 Version. International Conference on Harmonisation (ICH). Available online at: https://database.ich.org/sites/default/files/Q5E%20Guideline.pdf (Accessed May 14, 2026). 15988855

[20] European Medicines Agency. (2025). Guideline on Quality, non-clinical and Clinical Requirements for Investigational Advanced Therapy medicinal Products in Clinical Trials. European Medicines Agency. Available online at: www.ema.europa.eu/contact

[21] Department of Health TGA. (2005) ICH topic Q 5 E – Comparability of Biotechnological/ Biological Products. Available online at: https://www.tga.gov.au/resources/resources/international-scientific-guidelines-adopted-australia/ich-topic-q-5-e-comparability-biotechnological-biological-products (Accessed June 11, 2026).

[22] Therapeutic Goods Administration. (2025) Transition to new GMP Requirements for medicinal Products: A Notice about the Implications of Adopting the PIC/S Guide to GMP PE009-17. Available online at: https://www.tga.gov.au/resources/resources/user-guide/transition-new-gmp-requirements-medicinal-products (Accessed May 14, 2026).

[23] ISO. (2015). Cleanrooms and Associated Controlled Environments-Part 1: Classification of air Cleanliness by Particle Concentration. International Standard (ISO). Available online at: https://www.iso.org/standard/53394.html (Accessed May 14, 2026).

[24] ISO. (2017) ISO/IEC 17025:2017. Available online at: https://www.iso.org/standard/66912.html#:~:text=ISO%2FIEC%2017025%20is%20the,their%20testing%20and%20calibration%20results (Accessed May 19, 2026).

[25] Sin WX Jagannathan NS Teo DBL Kairi F Fong SY Tan JHL . A high-density microfluidic bioreactor for the automated manufacturing of CAR T cells. Nat Biomed Eng. (2024) 8:1571–91. doi: 10.1038/s41551-024-01219-1, 38834752

[26] Wang B Kanwar B Bowles-Welch AC Byrnes W Costa PC Filan C . Automated vertical wheel bioreactor integrated with process analytics for T-cell manufacturing. Cytotherapy. (2025) 27:1408–18. doi: 10.1016/j.jcyt.2025.08.007, 41032004 PMC13431691

[27] Mucha M Štach M Kaštánková I Rychlá J Vydra J Lesný P . Good manufacturing practice-grade generation of CD19 and CD123-specific CAR-T cells using piggyBac transposon and allogeneic feeder cells in patients diagnosed with B-cell non-Hodgkin lymphoma and acute myeloid leukemia. Front Immunol. (2024) 15:15. doi: 10.3389/fimmu.2024.1415328, 39192973 PMC11347927

[28] Monjezi R Miskey C Gogishvili T Schleef M Schmeer M Einsele H . Enhanced CAR T-cell engineering using non-viral sleeping beauty transposition from minicircle vectors. Leukemia. (2017) 31:186–94. doi: 10.1038/leu.2016.180, 27491640

[29] Luostarinen A Vuorela A Kerkelä E Patrikoski M Kotovuori A Koski J . Establishing a GMP-compliant manufacturing process and phase-appropriate analytics for early development of a FiCAR T-cell product with a novel CAR spacer. Sci Rep. (2025) 15:8093. doi: 10.1038/s41598-025-92736-9, 40057567 PMC11890757

[30] European Medicines Agency. (2014) Guideline on the Quality, non-clinical and Clinical Aspects of gene Therapy medicinal Products. Available online at: www.ema.europa.eu/contact

[31] European Medicines Agency. (2008) Guideline on Quality, non-clinical and Clinical Aspects of medicinal Products Containing Genetically Modified cells End of Consultation (Deadline for Comments). Available online at: www.ema.europa.eu/contact

[32] Therapeutic Goods Administration (2006). TGA Guidelines for Sterility Testing of Therapeutic goods about the Therapeutic Goods Administration (TGA). Available online at: http://www.ag.gov.au/cca (Accessed July 13, 2026).

[33] Office of Parliamentary Counsel. Gene Technology Regulations 2001. No. 106. Forrest: Office of Parliamentary Counsel (2025).

[34] O’Sullivan GM Philips JG Mitchell HJ Dornbusch M Rasko JEJ. 20 years of legislation – how Australia has responded to the challenge of regulating genetically modified organisms in the clinic. Front Med. (2022) 9:883434. doi: 10.3389/fmed.2022.883434 PMC9127347 35620726

[35] Office of the Gene Technology Regulator (OGTR). (2022) Guidance for Conducting human Clinical Trials Involving GMOs Australian Government Department of Health and Aged Care Available online at: https://www.ogtr.gov.au/sites/default/files/2022-10/guidance_for_conducting_human_clinical_trials_involving_gmos.pdf (Accessed May 14, 2026).

[36] Department of Health TGA. (2024) CTA scheme Forms. Available online at: https://www.tga.gov.au/resources/resources/forms/cta-scheme-forms (Accessed May 19, 2026).

[37] Department of Health TGA. (2014) Understanding the Common Technical Document (CTD). Available online at: https://www.tga.gov.au/resources/guidance/understanding-common-technical-document-ctd (Accessed May 19, 2026).

[38] ICH. (1995) Q5Bquality of Biotechnological Products: Analysis of the Expression construct in cells Used for Production of R-DNA Derived Protein Products Current Step 4 Version. International Conference on Harmonisation (ICH). Available online at: https://database.ich.org/sites/default/files/Q5B%20Guideline.pdf (Accessed May 14, 2026).

[39] ICH (2009) Guidance on Nonclinical Safety Studies for the conduct of human Clinical Trials and Marketing Authorization for Pharmaceuticals M3(R2) Current Step 4 Version. International Conference on Harmonisation (ICH). Available online at: https://database.ich.org/sites/default/files/M3_R2__Guideline.pdf (Accessed May 14, 2026).

[40] Department of Health TGA. (2019) Guidance 10: Adventitious Agent Safety of Medicines. Canberra:Therapeutic Goods Administration.

